# Whose voices? Patient and public involvement in clinical commissioning

**DOI:** 10.1111/hex.12475

**Published:** 2016-06-29

**Authors:** Alison O'Shea, Mary Chambers, Annette Boaz

**Affiliations:** ^1^ St George's, University of London & Kingston UniversityLondonUK

**Keywords:** health care, lay members, patient and public involvement, representation

## Abstract

**Aim:**

This paper aims to explore patient and public representation in a NHS clinical commissioning group and how this is experienced by staff and lay members involved.

**Background:**

Patient and public involvement is believed to foster greater public representativeness in the development and delivery of health care services. However, there is widespread debate about what representation is or what it should be. Questions arise about the different constructions of representation and the representativeness of patients and the public in decision‐making structures and processes.

**Design:**

Ethnographic, two‐phase study involving twenty‐four observations across two types of clinical commissioning group meetings with patient and public involvement, fourteen follow‐up interviews with NHS staff and lay members, and a focus group with five lay members.

**Results:**

Perceptions of what constitutes legitimate representativeness varied between respondents, ranging from representing an individual patient experience to reaching large numbers of people. Consistent with previous studies, there was a lack of clarity about the role of lay members in the work of the clinical commissioning group.

**Conclusions:**

Unlike previous studies, it was lay members, not staff, who raised concerns about their representativeness and legitimacy. Although the clinical commissioning group provides resources to support patient and public involvement, there continues to be a lack of clarity about roles and scope for impact. Lay members are still some way from constituting a powerful voice at the table.

## Introduction

There is a growing international interest in community participation in public sector services amid notions that partnership working increases the likelihood of meeting the population's needs and promotes community empowerment and equality.^1^ In the United Kingdom, all National Health Service (NHS) organizations have a duty to involve patients and the public in decision‐making around service planning, operation and proposals for changes.^1^, ^2^

Encouraging patient and public involvement (PPI) in decision‐making within health care is thought to be driven by the assumption that the public should have a role in shaping publically funded services.^3^ Also, that this involvement will result in more responsive services and improved health outcomes.^3^, ^4^

One area of contention around PPI in health care service development, and the focus of this paper, relates to representation. Representation warrants greater attention, because when it comes to making decisions there will always be a few who decide on behalf of others.^5^ Improving representativeness, including reaching seldom‐heard groups, is one of the rationales for PPI.^6^ PPI is believed to foster greater public representativeness in the development and decision‐making structures and processes of health care services. It is argued that lay people can provide a valuable service user perspective that is distinct from that of professionals.^7^ However, there are conflicting claims around what constitutes representation.^5^, ^8^

Typically, public representation in health care services is organized to provide feedback about the public's views and experiences of services. The processes through which public representation can occur include consultations, PPI in health‐specific interest groups and lay membership on trust boards and PPI committees. With increasing expectations for health care professionals and providers to show public accountability, these processes are also a means of achieving this. However, commitment to public accountability can, in practice, be rudimentary.^9^ The NHS 5‐Year Forward View^10^ sets out changes that are required to promote well‐being and prevent ill health, and argues for greater engagement with patients, carers and citizens. The report emphasizes the significance of developing stronger working partnerships with charitable and voluntary organizations. This is because of the information, advice and advocacy they provide, and their capacity to reach underserved groups. Whilst there is an expectation to include PPI in health care commissioning, NHS policies^10^, ^11^ do not provide concrete guidelines about the nature and extent of PPI in the process.^6^

This paper explores patient and public representation in two specific areas of a clinical commissioning group (CCG). CCGs are the product of structural changes within the NHS. From April 2013, commissioning powers shifted from the abolished Primary Care Trusts to CCGs. CCGs have responsibility for commissioning the majority of hospital and community services in health. This includes planned hospital admissions, emergency care, rehabilitation services, most community health services, mental health and learning disability services. There are 211 CCGs in total covering England.

### Representation

Different requirements and types of lay involvement have been widely debated as a means of achieving representativeness, depending on what it is that needs representation. In its narrow context, legitimate representation might require a representative to share a characteristic (e.g. gender, age, ethnicity) with the population they seek to represent: important in ensuring that the views and interests of minority groups do not become marginalized. This type of representation may be relevant where a range of public opinions are sought.^5^ However, characteristic‐sharing between a representative and those they represent may be unimportant when a representative is selected to act on behalf of another person or group of people for decision‐making purposes. In this case, it is responsiveness and accountability of the representative to those they represent which are important components.^5^

Questions about representativeness are often used in arguments for and against public involvement.^5^ Some professionals view public representation as too subjective and question the extent to which such representation can be afforded legitimacy.^8^, ^12^ Other professionals consider a subjective viewpoint as a valuable one that should not be discredited. To constrain the public's knowledge and subjectivities can be to exclude certain public groups.^8^

Patient and public representation also presents challenges for service commissioners. There is often lack of time and resources, interest among professionals and ‘knowledge of how to translate patient involvement into changes in health services’. Commissioners often face challenges in how they should achieve proper representation, where communities are diverse and there are many voices to be heard and reconciled.^6^

### Knowledge and skills

An area of tension around representativeness relates to the ordinary vs. extra‐ordinary status of lay people. On the one hand, as community representatives, lay people are required to be ordinary (that is, non‐experts) to understand the views and needs of the local community. On the other hand, they are expected to possess or develop the skills and knowledge deemed necessary by professionals to participate effectively. Representatives are required to possess negotiation skills, understanding of medical knowledge and an ability to see beyond their own personal experience,^13^ posing conflict with the ordinariness that representation entails.^13^, ^14^, ^15^, ^16^ This results in what Learmonth *et al*. describe as a ‘Catch‐22’. For effective public involvement in groups, ‘you have to be ordinary to represent the community effectively, but, if you are ordinary, you cannot effectively represent your community’.^15^ ^(p 106)^ In developing the particular skills required by health officials, lay people can become quasi‐professionals.^13^ They may support the interests of professionals or managers^17^ rather than the local community they represent, raising questions of legitimacy. Developing the required skills can also empower lay representatives to contribute in more meaningful ways.^12^

This paper explores CCG staff's approach to patient and public representation and the experiences of lay (public) people who participate in these processes. The study on which this paper is based is part of a wider project looking at PPI in an NHS CCG.

## Methods

This is an exploratory case study using ethnographic methods to investigate public representation in CCG board public and PPI reference group (RG) meetings.

The strength of the case study method is its ability to examine, in‐depth, a ‘case’ within its ‘real‐life’ context.^18^, ^19^ Ethnographic methods allow for the study of social interactions, behaviour and perceptions within groups or organizations. This permits using multiple methods of data collection such as observation, interviews and documentary evidence.^20^, ^21^ The purpose is to understand the phenomenon of interest from the perspective of those involved, accessing participant perceptions, experiences and social interactions in the context in which they occur.^22^, ^23^

This study was granted ethical approval on 14/02/14 by the East of Scotland Research Ethics Service.

### Data collection

The setting for this two‐phase case study is a large, diverse, inner city borough. Data collection took place over an 18‐month period, from February 2014 to August 2015.

#### First phase

Researchers conducted non‐participant observations of two types of meetings in which there was PPI: (i) CCG board public meetings, held monthly; (ii) PPI RG meetings, held bimonthly. CCG board public meetings were chosen because of being open to all members of the public without any form of membership required. RG meetings were chosen because the group specifically focused on PPI. The idea for the study emerged through discussion with the then lay chair of the RG who was interested in developing PPI in the local CCG.

- CCG board public meetings lasted 2½ hours and were open to members of the public to participate by asking questions and giving their views on issues under debate. Each meeting tended to focus on different aspects of the CCG's work, generating attendance from different members of the public. For this reason, researchers observed 14 of these meetings (approx. 35 hours). The CCG board comprised NHS managers, clinicians, a Healthwatch representative (Healthwatch is a consumer champion with statutory powers to ensure the voice of the consumer is heard throughout all aspects of health and care services in England) and two lay members, one with a remit for governance and the other for PPI.
- PPI reference group (RG) meetings lasted between 2½ and 3 hours. The group had been set up by the CCG a few months prior to the study commencing, primarily to support the development of PPI. Researchers observed ten RG meetings (approx. 30 hours) to note the progression of this relatively new group. The group comprised eleven lay (non‐CCG staff) members and five CCG staff. Lay members included five people and communities representatives (also referred to as ‘patient and public representatives’), two voluntary and community sector representatives, three locality representatives and the lay member chair of the RG. Staff members included a clinical lead, a PPI manager, an engagement manager, a CCG board member with a remit for PPI and an administrator.

Researchers’ field notes from observations of all meetings were entered onto a data collection tool (designed specifically for the study) for later analysis. The RG Terms of Reference, lay member recruitment documents and meeting papers were also collated for later analysis.

#### Second phase

Fourteen follow‐up interviews and a focus group were conducted to enable further exploration of issues around representation that had been noted during observations.

All lay members of the RG were invited to take part in a one‐to‐one interview and the focus group. Seven gave an interview and five took part in the focus group, with an overlap of three who took part in both. Lay members who did not agree to an interview gave reasons, which included lack of time and being away for a lengthy period. Three RG staff members took part in an interview, one of whom also sat on the CCG Board. Of the remaining two RG staff members, one was on long‐term sick leave and the other occupied an admin post.

The remaining four interviews comprised three with CCG board members and one with a member of public who regularly attended CCG board public meetings. Three further members of the public present at these meetings were invited to give an interview but either declined or did not respond to email invitations. Interviews lasted between 25 and 50 minutes. The RG focus group lasted almost 2 hours.

Interview and focus group questions were informed by observations of meetings and literature on lay representation. Questions were designed to explore participants’ perceptions of representation, and what representation involved. Interviews and the focus group were digitally recorded and transcribed verbatim.

### Data analysis

Interview and focus group transcripts and researchers’ observation notes underwent thematic analysis using a framework approach,^24^ which enabled systematic classification and analysis of data. Analysis was conducted initially using inductive methods to identify emergent themes within the data and then deductively, consistent with interview and focus group topic guides. This process was conducted independently by two team members across half of the data to identify emerging themes and then discussed. Once agreed, the remaining data were analysed and coded accordingly by one team member. Meeting papers and RG documents were used for reference purposes only, to draw on during analysis of interview, focus group and observation data.

### **Findings**

The term ‘lay member’ will be used in subsequent sections of this paper to refer to patients and other members of the public who participated in CCG board public meetings and RG meetings. ‘Staff member’ applies to CCG members of staff who sat on board public meetings and/or RG meetings (Table 1).

**Table 1:** Number and type of attendees at CCG and PPI RG meetings^a^

CCG board public meetings		PPI RG meetings	
Board members (staff)	*n* = 15	Staff members	*n* = 5
Board members (lay)	*n* = 3		
Members of public (non‐CCG staff)	*n* = 12	Lay members	*n* = 11

a Maximum number at any one meeting observed, although attendance numbers varied.

### Recruitment

To be eligible for recruitment to the RG, all members were required to live or work in the borough. Vacancies to join the group were advertised on the CCG's website, in GP surgeries and through voluntary sector and other organizations the CCG had links with. Application was through completing an application form or CV, and interview. Some lay members reported having taken part in a role‐play exercise and described the application process as having to ‘jump through hoops’.

The selection criteria included ensuring that different parts of the community would be represented, which otherwise might not. Recruitment documents described a requirement for different geographical areas of the borough to be represented by lay membership and capacity to identify with different groups of people, for example young/old, black minority ethnic groups and people with mental or physical health problems. In reality, whilst each identified geographical area had lay member representation, lay members tended to be semi‐retired or retired and most were white and described themselves as middle class.

Other recruitment criteria had included capacity to understand and represent the patient experience and to keep patients and the public informed of and involved in the work of PPI and the CCG. Interviews with staff members revealed less emphasis placed on some of these criteria. Networking and engaging with wider audiences to gain ideas and perspectives were valued, but not essential in lay members who were not representatives of a particular group. Other lay members who represented Healthwatch or a voluntary sector organization, however, were expected to have sound knowledge of that group, be aware of the associated issues and services, and have established networks and links with other organizations.

In contrast to the RG, CCG board public meetings provided an open forum where members of public could self‐select to attend. There was some overlap at the two meetings, with some RG lay members also attending CCG board public meetings, and with the RG chair who sat on the board as lay member for PPI. Two further lay members sat on the CCG board: one was a representative for Healthwatch and the other a lay member for governance. Recruitment to the board for the PPI and governance lay members had involved an open recruitment process. These posts were publicly advertised, and application was through CV and interview. Both lay members had previous experience of holding various corporate and governance roles in the CCG and/or the NHS more broadly.

### Knowledge and skills

Appointing different types of lay members meant that the RG benefited from a broad range of knowledge and skills. Staff members pointed out that discussion was key to RG lay membership, and possessing knowledge and skills enabled more effective contributions to discussion. Each lay member brought different and equally valuable views and experiences to the table:

It depends on what you're discussing as to how they can engage. And you draw on all the different bits of their experience to actually contribute into the discussion. (staff member, interview)

Providing support and training was also important in order for lay members to contribute to meetings effectively:

You know, you need to […] work through the thing ‐ ‘which do you understand, how can you contribute into this, what is the best way’, […] so when they come to the meeting then they are more empowered to understand what's going on and it becomes a proper dialogue. (staff member, interview)

Many lay members agreed that support and some training was useful to enhance their knowledge and understanding. They had requested training on particular areas of the CCG's work to facilitate this, for example, the commissioning process. Lay members commented that the range of pre‐existing skills, experiences and resources required, would exclude some members of the public from joining the group. They suggested people may not have, for example, IT skills and facilities, or financial resources to travel to meetings.

### What does representation mean?

The term ‘representation’ carried many different meanings for respondents. One staff member discussed representation in the context of lay participation in CCG‐organized community activities, patient and reference groups and board meetings:

You could have one patient turn up and they might be valid and very knowledgeable etc. but that wouldn't be very representative […]. Before you get true representation, […] you have to have good understanding of the population or patients. With that you can develop true representation. [The borough's] population is varied, diverse, not easy to get representation. (staff member, interview)

For RG lay members, views of representation were tied in with perceptions of the RG's role, which was unclear; representation was shrouded in uncertainty about what the group's role was. Lay members sought greater clarity about the role of the RG, what they were expected to achieve, and who they were representing and informing. They considered the RG's terms of reference did not make this clear:

[Member] referred to the terms of reference, stating uncertainty over what/who she is representing or informing as part of the RG, stressing that she felt unclear on this and uncomfortable. (researcher RG meeting observation notes)

Uncertainty was more apparent where lay members did not represent an organization or interest group; for lay members who did, the role was clearer. It was their responsibility to gain the views of the people they represented, ensure those views were heard and give feedback about the RG and the CCG's work. For one staff member:

The Healthwatch rep is a true representative because they join up what we do and what Healthwatch do […]. That person facilitates all the information flow either way […] and that is what I call the best, probably the most clear kind of representation. (staff member, interview)

A member of public who attended CCG board public meetings was concerned about patient and public representation on the board. They had a very particular view of representation relating to political characterizations of representation:

There's no elected patient reps on there whatsoever […]. (member of public, interview)

This member of public questioned the CCG's commitment to equality and democracy because neither board lay members nor staff members had been elected.

### Whose voice is being (or should be) heard?

During CCG board public meetings, when invited, the public would ask questions and give their views. On occasions, the public's questions related to personal experiences or views of health care services which at times were not welcomed. Due to meetings over‐running, many questions from the public would be responded to outside of and/or in writing after the meeting.

The slot allocated to patient and public questions was problematic:

I think it's better to have a couple of minutes after say half a dozen major agenda items than 10 minutes at the end […] otherwise […] someone like me might have put in 3 questions, [which] means by the time those are answered, nobody else gets a look in. (member of public, interview)

This same member of the public considered the meeting was for *informing* the public, not incorporating its views, and doubted whether they, or PPI more broadly, had any effect on the CCG's decision‐making.

RG staff members welcomed the personal perspective and broader community views and experiences that lay members could bring to meetings:

[Lay members] have personal experience […which gives] some insight into what it feels like to be a patient receiving or using a service that's been commissioned by the CCG. (staff member, interview)

Patient experience was important to RG staff and described as lay members’ own experiences as patients or users of health care services. They could draw on this information to contribute to RG discussion on services and service development. Staff emphasized that lay members should not represent a particular group of individuals solely on the basis of sharing particular characteristics:

If they have a mindset that ‘I can only represent white […] women who live in [the borough], then in some ways they shouldn't be there. (staff member, interview)

RG lay members, conversely, had concerns about the group not reflecting the community's diversity. During an observation, one lay member gestured around the room and commented:

Look at who makes up the representation ‐ middle class, middle aged, white

There were nods and murmurs of agreement around the table as the lay member continued that the group was:

Not actually representing the wider base of patients […] it is structurally unequal

These issues were raised during focus group and interview discussions as well as in RG meetings. Staff and lay members agreed:

No one group, let alone person, could represent the ideas and experiences of the whole of [the borough]. (staff member, interview)

I've actually lived in […] coming up for 50 years and in that time I've brought my kids up here and been to the same GP surgery all that time, so you do have a certain amount of accumulation. And that's really what it's about, it doesn't have to be 50 years, you know, if you've done some kind of involvement that gives you a feel for the community. (RG lay member, interview)

The experience of several years’ living in the locality, raising children, working and belonging to interest groups, exposing them to the wider community, earned validity to what otherwise might have been perceived a ‘too personal’ (and therefore invalid) perspective.

Lay members were asked whether they were treated as speaking on behalf of others. They questioned how they would know this as it had never been made clear to them, alluding to a lack of feedback about their input. One lay member commented:

Has the CCG ever really thought about us as representatives, rather than the reference group is a group they have to take into consideration. (RG lay member, focus group)

Lay members viewed the CCG as ticking boxes at times to demonstrate representation of particular groups. Another lay member emphasized:

The thing that really gets my goat is “the carer, the patient,” you know, I am the carer voice. Well you know there are different carers and many, many voices and that […] really makes me a bit worried […] but the attitude is “we've got a carer, they're all the same, it doesn't matter” […]. (RG lay member, focus group)

## Discussion

### True representation

One area of consensus among respondents in this study is that it is impossible to achieve true representation of a whole, diverse and large population. Yet the concept of true representation has different meanings for different people. Literature highlights the relevance of characteristic‐sharing;^5^ responsiveness and accountability;^5^ the subjective or patient experience^9^, ^11^ each as a means to achieving true or legitimate representation, depending on what is being debated or sought. The RG staff and lay members alike in this study viewed true or legitimate representation as representing a particular organization or group; understanding the community; and representing the values of the community. However, one of the main concerns of RG lay members was the extent to which the group constituted legitimate representation, particularly in relation to characteristic‐sharing with those they sought to represent. It is notable that this finding was more strongly evidenced during earlier observations, when the RG was less well established. One possible reason for this is that lay members later viewed legitimate representation as networking and linking with other community groups, and as ‘expert by experience’.^25^ RG staff were not concerned about the representativeness of lay members’ characteristics. This may in part be related to there being a number and variety of PPI activities in place, indicating the CCG was developing multiple methods to achieve patient and public representation. Similarly, the individual patient experience that appeared to be welcomed more by RG staff than CCG board staff might be explained by particular views possibly held by RG staff members relating to their job description to support PPI and the RG.

A second concern of lay members around their legitimacy related to the knowledge, skills and resources they possessed and which the CCG required in order for them to join the RG. Possessing knowledge, skills and resources arguably placed lay members into the ‘extraordinary’ category of Catch‐22 mentioned earlier and was a double‐edged sword for lay members because it also accentuated the exclusive nature of the group which already lacked diversity in its narrower sense.

### Expert by experience

Previous studies^8^, ^12^, ^17^ have found that staff or professionals tend to question the legitimacy of public representatives (often based on non‐characteristic‐sharing between representatives and those they claim to represent) and often dismiss the view of patient representatives as not typical.^26^ Public representatives, on the other hand, consider that their status as members of the public, their skills, experience and knowledge of the community make them sufficiently eligible to represent the wider public.^8^ RG lay members acknowledged their contribution as experts by experience, not least by a number of years living, working, raising families and using services in the local area. However, they questioned whether being an expert by experience, derived in this way, was sufficient to represent diverse groups within the community. What might be the possible implications if patient and public representation was framed in terms of being an expert by experience rather than by characteristic‐sharing? Much would depend on what expert by experience involves. It would seem that, at best, if representatives network with and feedback on the experiences of seldom‐heard groups, this would be one means of these groups having a voice. However, arguably the voice would be stronger coming from a representative with shared characteristics of the group they represent because of their lived experience or knowledge.

### Supported or tokenistic patient and public representation?

Statutory authorities are viewed as continuing to control the rules of public engagement, for example the nature and level of engagement; type of public representation; and arrangements for meetings including form and content.^1^, ^26^, ^27^ This study reveals that during CCG board public meetings, public input tended to be somewhat restricted to questions deemed relevant to the agenda items under discussion. On occasions, public voices were closed down and questions on non‐agenda items unwelcomed. Together with their questions being responded to outside of the meetings, this suggests controlled and a lack of – or tokenistic – public input. In order for the public's voices to be heard publicly, greater allocation of time to the public slot was needed during these meetings.

Consistent with previous studies,^14^, ^17^ there was at times uncertainty among lay members around the role of the RG and therefore their representative role, further exacerbated by not having received feedback from the CCG about their work. This uncertainty was less frequently expressed by lay members towards the end of researcher observations, which may suggest they had gained clearer understanding as the RG developed. However, a PPI rationale or a role which is unclear or has marginal impact ‘can easily slip into tokenism’.^28^ ^(p e44)^ Where involvement is tokenistic, patients and the public become disengaged and less involved – a process described as a ‘vicious circle’. By contrast, where engagement is not tokenistic, a good or ‘virtuous’ circular process can lead to improved involvement.^27^

RG lay members had at times viewed their involvement as a tickbox exercise for the CCG. Despite appearing disillusioned on occasions, they did not seem to become disengaged and less involved as the vicious circle describes. Neither was there an obvious improvement in their involvement – to suggest a virtuous circle – as they had shown from the early stages of the RG, and continued to show, commitment to developing PPI activities and supporting the work of the CCG.

The group had not received feedback about its contributions, resulting in difficulty assessing whether or how it had had an impact. Researchers did observe developments in the area of PPI itself and improvements to some CCG initiatives in which there had been PPI, for example the RG was consulted on the content and wording of a survey aimed at members of the public. However, it remained unclear to researchers as well as to lay members how and where PPI fed into the CCG at a more strategic level in terms of decision‐making processes. This finding corresponds with previous research^27^, ^29^ where PPI impact on design, evaluation and reconfiguration of health care services has been identified,^29^ yet PPI impact on strategic decision making has been difficult to determine.^27^, ^29^

Feedback from the CCG to RG lay members might have provided greater clarity on their role and on the group's achievements. A collaborative approach between staff and lay members from the outset of the RG may also have facilitated greater understanding of expectations and accountability in relation to the group's role. Moreover, evaluation of the CCG's PPI could improve understanding around PPI effectiveness. Using an analytic framework that looks at whether PPI approaches are moral (does everyone have a voice?); whether they are approached methodologically (has quality been improved?); and the policy imperative (has PPI been implemented according to policy?)^27^ could provide valuable insight into areas for further development, good practice and impact. However, effective evaluation of PPI is not without its challenges: it could take several years to measure outcomes.^6^

Good practice of patient and public representation has emerged through this study. The CCG provided resources to support PPI: staff in the form of a PPI lead; manager and admin support; and a board member with a remit for PPI. The PPI RG met bimonthly, with a meeting room and refreshments provided. Training relating to the work of the CCG had also been delivered to lay members. These resources facilitated the involvement of lay members in the RG. However, whilst such resources might be considered good practice in terms of being an important component to achieving maximum PPI effectiveness, lay members considered that good practice could only be defined as such if service improvement was evidenced as an outcome.

### Lessons learned from this study

The research team was impressed with the general interest in the study and the straightforwardness of recruiting RG members. The willingness to participate may have been as a result of the researchers having become familiar to members through observing RG meetings over several months.

The data collected were from two types of PPI within the CCG. These were very specific activities, and it is important to keep in mind that PPI operated in other types of CCG activities and groups. Therefore, the study has gained a particular view of patient and public representation, which might not reflect PPI across the whole CCG.

Using a case study approach and different methods of data collection allowed for triangulation across the data sets, contributing to the overall richness of the study outcome. For example, some of the RG lay member interviewees also attended public meetings which helped give a broader view of the public's experience.

For this study to acknowledge the contribution of case study design is of itself ‘not new learning’ but rather reinforces what is already known.

There are of course limitations to using case study design as findings cannot be generalized. However, that was never the intention here. Given the nature of the study site – a large, inner city borough with a diverse population and different social class‐affluence – it is possible that findings would have been different if the study had taken place in a less densely and diversely populated, rural area. A future study might explore PPI in other CCGs to strengthen representativeness and enable a comparative analysis. Future research might also want to explore whether outcomes are more positive for seldom‐heard groups as a result of patient and public representation, and what type of representation best facilitates this.

## Conclusion

This case study provided useful insights into how PPI is integrated into CCG activity and highlighted some areas of good practice, for example the CCG provided resources to support PPI.

Of particular note emerging from the study was that the issue of legitimate representation was raised by RG lay members and not CCG staff which would appear to differ from other work.^8^, ^12^, ^17^ Lay members were concerned about the nature of the representativeness and how they could best represent the diverse community. This for them was a real issue of concern as was clarity about their role overall and their impact. Whilst this study adds to the knowledge base about patient and public representation in health care commissioning, it does raise further questions that warrant investigation. For example, what is legitimate knowledge; how can patient and public representation contribute to commissioning decision‐making; and how can the value of their representation be captured?

## Source of funding

The research was supported by the National Institute for Health Research (NIHR) Collaboration for Leadership in Applied Health Research and Care (CLAHRC) (award no.: NIHR, CLAHRC‐2013‐10022) South London, at King's College Hospital NHS Foundation Trust, and the Faculty of Health, Social Care and Education, St George's, University of London & Kingston University. The views expressed are those of the authors and not necessarily those of the NHS, the NIHR or the Department of Health.
